# Novel Insights into Torrefacto and Natural Coffee Silverskin: Composition, Bioactivity, Safety, and Environmental Impact for Sustainable Food Applications

**DOI:** 10.3390/foods14193388

**Published:** 2025-09-30

**Authors:** Ernesto Quagliata, Silvina Gazzara, Cecilia Dauber, Analía Rodríguez, Luis Panizzolo, Bruno Irigaray, Adriana Gámbaro, José A. Mendiola, Ignacio Vieitez, María Dolores del Castillo

**Affiliations:** 1 Food Science and Technology Department, School of Chemistry, Universidad de la República (UdelaR), Av. General Flores 2124, Montevideo 11800, Uruguay; equagliata@fq.edu.uy (E.Q.); silvigazzara@gmail.com (S.G.); cdauber@fq.edu.uy (C.D.); arodriguez@fq.edu.uy (A.R.); apanizzo@fq.edu.uy (L.P.); birig@fq.edu.uy (B.I.); agambaro@fq.edu.uy (A.G.); 2 Instituto de Investigación en Ciencias de la Alimentación (UAM–CSIC), Nicolás Cabrera 9, 28049 Madrid, Spain; j.mendiola@csic.es

**Keywords:** coffee silverskin melanoidins, torrefacto, antioxidant, safety, clean label sustainable ingredient, circular economy, life cycle assessment

## Abstract

Coffee silverskin (CS), the principal solid by-product from coffee roasting, is a promising raw material for sustainable food applications aligned with circular economy principles. Due to its high flammability at roasting temperatures, effective management of CS is not only an environmental but also a safety concern in coffee processing facilities. To the best of our knowledge, this is the first study evaluating the chemical composition, bioactivity, safety, and environmental impact of torrefacto (CT) and natural (CN) coffee silverskin. CT (from Arabica–Robusta blends subjected to sugar-glazing) and CN (from 100% Arabica) were characterized in terms of composition and function. Oven-dried CT showed higher levels of caffeine (13.2 ± 0.6 mg/g vs. 8.7 ± 0.7 mg/g for CN), chlorogenic acid (1.34 ± 0.08 mg/g vs. 0.92 ± 0.06 mg/g), protein (18.1 ± 0.2% vs. 16.7 ± 0.2%), and melanoidins (14.9 ± 0.3 mg/g vs. 9.6 ± 0.2 mg/g), but CN yielded more total phenolics (13.8 ± 0.6 mg GAE/g). Both types exhibited strong antioxidant capacity (ABTS: 48.9–59.2 µmol TE/g), and all oven-dried samples met food safety criteria (microbial loads below 10^2^ CFU/g, moisture 7.9%). Oven drying was identified as the most industrially viable, ensuring preservation of bioactives and resulting in a 19% lower greenhouse gas emissions impact compared to freeze-drying. Sun drying was less reliable microbiologically. The valorization of oven-dried CT as a clean-label, antioxidant-rich colorant offers clear potential for food reformulation and waste reduction. Renewable energy use during drying is recommended to further enhance sustainability. This study provides scientific evidence to support the safe use of coffee silverskin as a novel food, contributing to regulatory assessment and sustainable food innovation aligned with SDGs 9, 12, and 13.

## 1. Introduction

**Figure 1:**
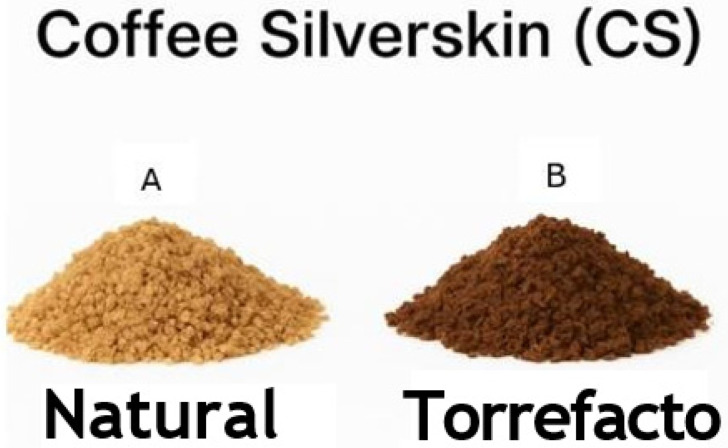
Natural (**A**) and torrefacto (**B**) coffee silverskin.

Coffee is currently produced in more than 50 countries, with over three billion cups consumed daily, generating annual revenues exceeding 200 billion USD [1]. During the processing of coffee to obtain roasted beans, approximately 90% of the harvested product—the coffee cherry—is lost, which generates by-products that can negatively impact the environment if not properly managed [1]. Among these by-products is coffee silverskin (CS), which accounts for approximately 5–6% of the green bean’s weight and is the only solid residue produced during roasting process. Globally, coffee roasting generates approximately 200,000 tons of CS per year as a by-product [2]. CS is available in two forms: natural (Figure 1A), obtained when no sugar is added during roasting, and torrefacto (Figure 1B), a traditional roasting method used mainly in some Latin American and Southern European countries, where sugar (typically 10–20%) is added during the final stage of roasting. This induces caramelization and a shiny coating on the beans, promoting the formation of Maillard-derived compounds and altering the silverskin composition [3,4].

The valorization of such by-products, including CS, presents an opportunity to promote more sustainable practices within the coffee industry, supporting the circular bioeconomy and environmental protection. Improper disposal of these materials can lead to pollution due to their organic content [5,6,7].

Coffee is derived from over 130 species, but only two are widely accepted for human consumption: Coffea arabica (Arabica) and Coffea canephora (Robusta). While single-origin coffee roasting has gained popularity, most commercial coffee products still rely on blends of different origins. Differences in the chemical composition of Arabica and Robusta beans influence not only their sensory properties but also their health effects (Table 1). Arabica coffee, which contains higher levels of carbohydrates, lipids, and compounds such as 3-feruloylquinic acid, offers a smoother flavor profile. In contrast, Robusta coffee, which is richer in caffeine, proteins, and chlorogenic acids, tends to be more bitter and exhibits distinct physiological benefits [8]. These differences also extend to the composition and properties of silverskin, the by-product generated during roasting [9].

Recent studies have highlighted the potential of coffee silverskin as a food supplement, due to its nutritional value, including a considerable protein content (14–19%), high dietary fiber levels (up to 55%), and a low fat content (<4%) [1,11,12]. In addition, it has been associated with health-promoting properties, such as anti-inflammatory, antidiabetic, and cholesterol-lowering effects, attributed to the presence of bioactive compounds like caffeine and chlorogenic acid, both known for their antioxidant activity [13,14]. On average, CS contains approximately 31 mg/g of caffeine and 10–20 mg/g of chlorogenic acid, depending on the coffee variety and extraction conditions [7]. Bioactive peptides with potential applications in the treatment of diabetes and its complications have also been identified [14].

Moreover, melanoidins, structurally complex, colored compounds formed during the roasting of coffee silverskin via the Maillard reaction, exhibit additional functional properties such as metal-chelating capacity, preservative effects, and the ability to interact with volatile aroma compounds, which could enhance food quality [15,16]. These structures have also demonstrated antioxidant, antimicrobial, and anti-inflammatory activities, as well as the capacity to increase intestinal motility [17], positioning them as promising functional food ingredients.

However, it is important to consider potential adverse effects related to Maillard and caramelization reactions during roasting, particularly the formation of undesirable compounds such as 5-hydroxymethylfurfural (HMF) and acrylamide, both of which have been identified as potentially toxic and carcinogenic [18,19]. Since the levels of these compounds may vary depending on the degree of roasting and the coffee species, a comprehensive evaluation of coffee silverskin as a food ingredient is essential, balancing its nutritional benefits against possible health risks [19,20]. Data refer only to coffee silverskin obtained by natural roasting are available. HMF levels below the LOQ and acrylamide values of 152 µg/kg and 161 µg/kg for Arabica and Robusta samples, respectively, were reported [9]. Acrylamide content of coffee silverskin extract itself was quantified at 11.42 µg/L, approximately ten times lower than the values typically observed in brewed coffee beverages (175–263 µg/L, FDA). These concentrations remained well below the European Commission’s indicative values for roasted (450 µg/kg) and instant (900 µg/kg) coffee, as well as for biscuits (500 µg/kg), suggesting that while silverskin contributes to acrylamide formation during baking, its levels remain within acceptable safety margins [21]. Moreover, Acrylamide has been detected in coffee silver skin (CS) at levels ranging from 24 to 161 µg/kg, depending on the sample type (arabica, canephora, or industrial pellets). These concentrations are lower than those typically reported for roasted coffee beverages, but their presence still requires consideration for potential food applications. Although acrylamide is classified by the International Agency for Research on Cancer (IARC) as a probable human carcinogen, the levels found in CS are within ranges observed for other cereal- and coffee-derived by-products, suggesting that its inclusion as a functional food ingredient remains feasible if properly monitored and controlled [9,22]. In this regard, studies assessing the chemical composition, toxicity, and biological activity of aqueous extracts from natural silverskin support its potential safety in food applications [23,24,25].

According to the FAO’s food waste management hierarchy pyramid, strategies that prioritize the reduction, reuse, and recycling of food and its by-products should take precedence over disposal [26]. This approach underscores the opportunity to valorize coffee silverskin instead of incinerating it, aligning with sustainable and health-promoting practices. However, its classification as a “novel food” in the European Union [27] means that further studies are required before it can be authorized as a food ingredient. In line with current global priorities, sustainable decision-making is essential in the design, production, and management of silverskin-derived products, supported by environmental impact assessments through Life Cycle Analysis [28].

Moreover, improper management of coffee silverskin, whether through inadequate storage or incineration, poses significant environmental hazards, including increased fire risk at industrial facilities. Such practices conflict with the FAO’s food waste hierarchy and global sustainability priorities, as they lead to the loss of valuable bioactive compounds that could otherwise be safely and effectively valorized in food applications, delivering benefits to both the industry and consumers. This underscores the urgent need to develop safe, functional, and sustainable strategies for the valorization of coffee silverskin as a novel food ingredient.

This study aims to provide comprehensive insights into the chemical composition, antioxidant capacity, safety, and environmental impact of torrefacto and natural coffee silverskin to support their sustainable reuse as functional food ingredients. In doing so, it seeks to contribute to the United Nations Sustainable Development Goals, particularly SDG 9 (Industry, Innovation and Infrastructure), SDG 12 (Responsible Consumption and Production), and SDG 13 (Climate Action), by promoting the valorization of coffee silverskin as an underutilized resource that can be transformed into a functional ingredient and serve as a model of circular economy within the food industry. While its use as a novel food in the EU still requires further research and regulatory evaluation, this by-product demonstrates high potential for waste reduction, cleaner production, and improved environmental sustainability. Advancing research in this field not only offers opportunities for the coffee sector but also generates broader benefits for consumers and the environment.

## 2. Materials and Methods

All reagents were analytical grade and were purchased from Sigma–Aldrich Inc. (St. Louis, MO, USA).

### 2.1. Material Collection and Pretreatment

**Figure 2:**
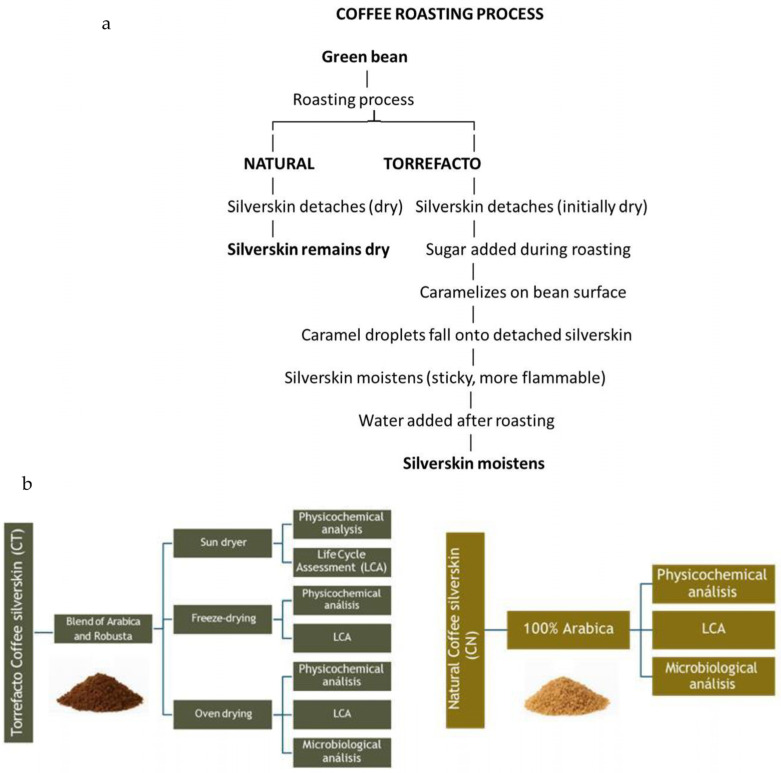
Silverskin from natural (CN) and torrefacto (CT) coffee: (**a**) flow during roasting, and (**b**) analyses performed.

Coffee silverskin was provided by Nestlé (Uruguay). In natural roasting, it detaches early and remains dry, whereas in torrefacto roasting sugar is added after detachment, forming caramel on the bean and releasing droplets that moisten the silverskin, increasing stickiness and flammability. To reduce this risk, water is applied before collection (Figure 2a). Two types of silverskin were obtained: torrefacto (CT, a Brazilian Arabica–Robusta blend with ~55–60% Robusta) and non-torrefacto (CN, exclusively Arabica from diverse origins: Brazil, Colombia, Costa Rica, Sumatra, Peru, and Guatemala) (Figure 2b). Torrefacto blends typically combine Arabica and Robusta, reflecting both historical and technological drivers, as sugar roasting was initially used to mask negative attributes in Robusta and remains mostly marketed as blends rather than single origins [29,30]. Consequently, differences between CN and CT silverskin reflect both origin and roasting, and cannot be attributed to a single factor. Because water is added during glazing for safety, CT required drying pretreatment.

Three different drying methods were evaluated for CT: oven drying (model DHG-9053A) at 40 °C for 24 h, freeze-drying (model BK-F010P) for 72 h, and indirect sun drying for 5 h (ambient temperature ranged between 9.0 °C and 17.8 °C, with sun irradiance between 127.5 W/m^2^ and 558.3 W/m^2^; the maximum temperature reached was 79.1 °C). The indirect solar dryer consists of a drying chamber and a 1.8 m^2^ solar thermal collector. At the inlet of the collector, there is a pump that injects air to be heated and subsequently feeds the chamber, which has an approximate volume of 0.1 m^3^. Since the collector has a large capture surface compared to the small volume to be heated, the air temperature (cp = 1000 J/kg·K) increases significantly inside the chamber. The final moisture contents achieved were 7.89 ± 0.52% (oven drying), 0.21 ± 0.02% (freeze-drying), and 18.22 ± 0.47% (sun drying), respectively. Although the maximum air temperature in the sun drying exceeded that of the oven drying, the higher ambient humidity and shorter effective drying period resulted in higher final moisture content in the sun-dried samples. It is worth noting that, under Uruguayan summer conditions, daily temperature fluctuations are pronounced, which may have further influenced the drying dynamics and moisture retention. All dried silverskin samples (CT and CN) were stored at 4 °C until further analysis.

### 2.2. Proximate Composition of Silverskin

To characterize both types of silverskin, moisture content (AOAC 23.003), protein (AOAC 955.04), lipid content (AOAC 991.36), ash (AOAC 920.153), and total fiber (AOAC 985.29) were determined. Carbohydrate content was calculated by difference, subtracting the percentages of lipids, proteins, ash, and total fiber from 100%. All analyses were performed at least in triplicate.

### 2.3. Silverskin Characterization and Properties

#### 2.3.1. Color Measurement

Color was determined using a colorimeter (Minolta CR-300) to measure the CIELAB parameters: L* (lightness, from black to white), a* (red-green axis), and b* (blue-yellow axis). Ten measurements were taken per sample, and the average values were calculated.

#### 2.3.2. Total Phenolic Content Determination

Phenolic content and antioxidant activity were determined through extraction using an 80:20 ethanol/water mixture, following the procedure described by Dauber et al. [31]. Briefly, 100 mg of silverskin was mixed with 10 mL of the solvent, vortexed for 1 min, magnetically stirred for 15 min, sonicated for 10 min, and then centrifuged at 6000 rpm for 10 min. The supernatant was filtered through a 0.45 µm filter and diluted 1:2 with distilled water before analysis.

Total phenolic content was measured using the Folin–Ciocalteu method as described by Singleton et al. [32] and modified by Fernandez-Fernández et al. [33], employing a gallic acid calibration curve (0.05–1.0 mg/mL). In a microplate, 10 µL of extract or standard solution was mixed with 200 µL of 20% (*w*/*v*) Na_2_CO_3_ solution. After 2 min, 50 µL of Folin’s reagent (diluted 1:5) was added, and the mixture was incubated in the dark for 30 min. Absorbance was measured at 750 nm using a Multiskan Go spectrophotometer (Thermo Fisher Scientific, Waltham, MA, USA). Results are expressed as mg gallic acid equivalents (GAE) per gram of silverskin.

#### 2.3.3. Antioxidant Capacity (ABTS)

Antioxidant capacity was assessed using the ABTS radical cation decolorization assay, according to Re et al. [34], with modifications by Fernández-Fernández et al. [33], using Trolox as the standard. An activated 7 mM ABTS solution was diluted with water until an absorbance of 0.7 at 750 nm was reached. In a microplate, 10 µL of extract or standard was mixed with 190 µL of the ABTS working solution. After 10 min of reaction, absorbance was measured at 750 nm using the Multiskan Go spectrophotometer. Results are expressed as µmol Trolox equivalents per gram of silverskin.

#### 2.3.4. Determination and Characterization of Melanoidins

The study of melanoidins was conducted based on the technique reported by Iriondo DeHond et al. [1] and Rodriguez et al. [35].

##### Extraction and Yield Evaluation of the Extract

Extraction was performed using distilled water at a concentration of 50 mg/mL of coffee silverskin, heated to 100 °C for 10 min, followed by filtration through a 250 µm filter. The extraction yield was evaluated by freeze-drying a measured volume of the extract.

##### Color Evaluation by Reflectance Spectrophotometry

The color of the extract was measured in a quartz cuvette against a white background using a reflectance spectrophotometer (CM-2300d) in the CIELAB color space. The parameters L* (lightness), a* (red-green axis), b* (yellow-blue axis), chromaticity (C*ab), and hue angle (h*ab) were determined.

##### Color Evaluation by Absorbance Spectrophotometry

Absorbance was measured at 420 nm in extract solutions at 2 mg/mL concentration. The color intensity of the sample was quantified using a calibration curve of absorbance at 420 nm with Class IV Caramel Colorant (INS 150d, DURYEA^®^ DP from Ingredion) tested at concentrations of 0.7 mg/mL and 7 mg/mL. Extract concentrations were expressed as mg equivalent of Class IV Caramel Colorant per gram of extract. Additionally, UV-Vis spectra of the extracts and the caramel colorant were recorded between 200 and 800 nm.

##### Molecular Weight Distribution of Melanoidins

Molecular weight distribution was analyzed by size exclusion chromatography (SEC-HPLC) according to Rodriguez et al. [35], using an Agilent Technologies 1260 Infinity HPLC system (Agilent Technologies, Santa Clara, CA, USA) equipped with a BioSep-SEC-S 3000 column (300 × 7.80 mm). The mobile phase consisted of phosphate buffer (pH 6.8, 50 mM) with 0.5% SDS, at a flow rate of 1 mL/min. Detection was carried out at 280 nm, 370 nm, and 420 nm. Injection volume was 20 µL. Molecular weight standards used were Blue Dextran (2000 kDa), BSA (66 kDa), and lysozyme (14.3 kDa). Samples were prepared at 2 mg/mL and standards at 1 mg/mL.

##### Quantification of Melanoidins by Molecular Weight

Melanoidin content was quantified by separating high molecular weight (>10 kDa) and low molecular weight (<10 kDa) fractions through ultrafiltration of undiluted extract using centrifugal filter tubes with a 10 kDa cut-off (Amicon Ultra, Millipore, Burlington, MA, USA). Two consecutive washes of the filtrate with 2 mL ultrapure water each were performed, followed by centrifugation. The melanoidin fractions were quantified gravimetrically after freeze-drying each fraction.

##### Antioxidant Capacity of Melanoidin Fractions

The antioxidant capacity of the melanoidin fractions was determined following the method described in Section 2.3.3.

#### 2.3.5. Determination of Caffeine and Chlorogenic Acid Content

Four different extraction methods reported in the literature were evaluated for the extraction of caffeine and chlorogenic acid, selecting the method that yielded the highest content of both compounds.

The first method, developed by De la Cruz et al. [17], used a solution of 50 g/L coffee silverskin in water. This solution was stirred for 10 min at 100 °C and then filtered.

The second method, described by Del Rio et al. [36], involved weighing 500 mg of coffee silverskin and adding 5 mL of 1% aqueous formic acid. The mixture was subjected to an ultrasonic bath for 30 min, and then heated with agitation at 70 °C for 1 h. Afterwards, it was centrifuged at 3500 rpm for 10 min, the supernatant collected, and the procedure repeated. The aqueous extracts were combined and filtered through a 0.45 µm filter.

The third method, according to Bessada et al. [37], consisted of adding 25 mL of a 1:1 ethanol-water mixture to 0.5 g of sample, followed by heating at 40 °C and stirring for 1 h. The mixture was then centrifuged at 4500 rpm for 10 min and filtered through a 0.45 µm filter.

The fourth method, reported by Martuscelli et al. [38], involved adding 10 mL of a 70:30 methanol-water mixture to 1 g of coffee silverskin. The sample was vortexed for 1 min, centrifuged at 4000 rpm for 10 min, and the supernatant recovered and filtered through a 0.45 µm filter.

Caffeine and chlorogenic acid detection was performed by HPLC (Shimadzu Model 20A) following Mirón-Mérida et al. [39]. A quaternary pump coupled to a diode array detector was used, with a Macherey C18 column (250 × 4.6 mm, 5 µm particle size), flow rate of 1 mL/min, and detection at 280 nm. The mobile phase consisted of a 3% acetic acid aqueous solution (solvent A) and acetonitrile (solvent B), starting at 100% A, gradually increasing B to reach 24% over 15 min, then maintained at that ratio for the remainder of the run. Quantification was performed using calibration curves prepared with caffeine and chlorogenic acid standards.

Among all the solvents tested, the 1:1 ethanol-water mixture was selected, as it provided the highest content of caffeine and chlorogenic acid compared to the other methods evaluated (see Table 2).

#### 2.3.6. Determination of Hydroxymethylfurfural (HMF) Content

The extraction of HMF was carried out following the method described by Rufián-Henares et al. [40]. Five hundred milligrams of coffee silverskin were weighed, and 5 mL of ultrapure water was added. The mixture was vigorously shaken for one minute, then clarified by adding 0.250 mL of Carrez solution I and 0.250 mL of Carrez solution II. The solution was centrifuged at 4500× *g* for 10 min at 4 °C, the supernatant separated, and the extraction was repeated twice more, adding 2 mL of ultrapure water each time. The supernatants from all three extractions were combined. The HMF content was determined by HPLC using a 1260 Infinity system (Agilent Technologies, Santa Clara, CA, USA) equipped with a diode array detector. A reversed-phase C18 column (Jupiter, Phenomenex, 250 × 4.6 mm, 300 Å, 5 µm) was used, with a flow rate of 1 mL/min and detection at 285 nm. The mobile phase consisted of ultrapure water and methanol in a 90:10 ratio. Quantification was performed using a calibration curve prepared with HMF standard.

#### 2.3.7. Life Cycle Assessment

The environmental impact of the three drying processes was assessed using Life Cycle Assessment (LCA) with the SimaPro 8 software (Pré Consultants B.V, Amersfoort, The Netherlands). The International Reference Life Cycle Data System (ILCD) method was selected for impact calculation, following the ILCD 2011 Midpoint+ approach (version 1.08) with EU27 2010 equal weighting. The comparison of drying methods was based on their final yield, with 100 g of dried pigment chosen as the functional unit (FU) to standardize environmental impact categories. The system boundaries focused exclusively on the drying stage, applying a gate-to-gate approach that excluded upstream and downstream processes related to raw material acquisition or subsequent processing. This approach allows for a direct assessment of the sustainability and environmental footprint of each drying method.

#### 2.3.8. Microbiological and Toxicological Analysis

Analyses for Ochratoxin A (AOAC 991.45) [41], mesophilic aerobes (ISO 4833-2013) [42], fungal and yeast counts [43] were carried out. Results were compared with relevant local regulations.

### 2.4. Statistical Analysis

All analytical methods were performed at least in triplicate. The data obtained for each method were analyzed using analysis of variance (ANOVA) and Tukey’s test to determine significant differences between the drying methods as well as between the dried silverskin samples. Statistical analyses were conducted using Infostat version 2020e.

## 3. Results and Discussion

### 3.1. Physicochemical Analysis

Regarding the comparison of drying methods for torrefacto coffee silverskin (CT), the goal was to achieve a moisture content below 8%, according to criteria of Ballesteros et al. [44]. The final moisture contents obtained were: 7.89 ± 0.52% for oven drying, 0.21 ± 0.02% for freeze-drying, and 18.22 ± 0.47% for sun drying. The freeze-drying method yielded the lowest moisture content; however, it requires more time and is less available at the industrial level due to its higher costs.

Table 3 shows the characterization values of the different silverskin samples: CT dried by the three processes (freeze-drying, oven drying, and indirect sun drying) and natural silverskin (CN). Although the origin of the CS is indeed different, the characterization of values remains valid as it provides useful insight into the influence of both production method and raw material origin.

As expected, dietary fiber was the predominant component across all samples, which supports its potential use as a functional food ingredient. Extensive research has demonstrated that sufficient dietary fiber intake confers numerous health advantages, including the promotion of a balanced gut microbiota and a reduced risk of colorectal cancer, type 2 diabetes, and cardiovascular disease [45,46]. In line with this, the World Health Organization (WHO) recommends a minimum daily intake of 25 g of natural dietary fiber for adults [47]. This target may be met through various strategies, including the reformulation of food products to incorporate fiber-rich materials such as coffee silverskin. The total dietary fiber content observed in this study aligns with values previously reported by Borrelli et al. [48], who measured an average of 67.3% (dry basis) in three samples of 100% Arabica silverskin, and Napolitano et al. [49], who found 69.2% (wet basis) in Robusta silverskin. Conversely, the protein content in the present samples was below the typical reported range of 16–18% [6]. Ash and lipid levels were consistent with those documented in earlier studies [37,44,50].

It was also expected that silverskins from the glazing process (CT) would not show significant variations in values among the three drying methods. However, since the torrefacto silverskin comes from a blend of Robusta and Arabica coffee species, it was expected to have a higher caffeine and chlorogenic acid content than CN, which comes from 100% Arabica coffee. The caffeine values align with those published by Iriondo DeHond et al. [23], who obtained more than double the caffeine content for Robusta coffee (53.3 mg/g) than for Arabica (24 mg/g).

The total phenolic content (TPC) did not show significant differences (*p* > 0.05) between the different silverskins and the values obtained were slightly higher than the range reported by Bessada et al. [37], who studied six samples of silverskin from different origins with TPC values ranging from 5.0 to 18.3 mg GAE/g. The samples from Brazil and Indonesia stood out for their higher TPC and antioxidant activity, which suggests that these parameters might be significantly related to the geographical origin of the coffee beans. The antioxidant capacity determined by the ABTS method was significantly higher (*p* < 0.05) in freeze-dried and oven-dried CT samples, with values exceeding those reported by Ballesteros et al. [44] (using DPPH assay) and Aroufai et al. [51] (using ABTS). The latter found significant differences in antioxidant capacity between Arabica and Robusta, with Robusta showing higher values, consistent with our results where the antioxidant capacity of CN was lower than those of CT. In addition, this difference can be attributed to the different melanoidin content between CT and CN.

Furthermore, CT (a blend of Arabica and Robusta) showed higher protein content than CN (100% Arabica) consistent with literature [52]. The caffeine and chlorogenic acid percentages were higher in CT compared to CN, which is also expected since the glazing process includes approximately 55–60% Robusta coffee, which has a higher content of both compounds. The lower caffeine content in CT sun-dried silverskin is likely related to its higher residual moisture and to potential microbial losses due to extended outdoor exposure.

Total phenolic content (TPC) results showed no significant differences (*p* > 0.05) among the three drying methods. However, considering the values obtained for caffeine and chlorogenic acid (Table 3), oven drying could be the preferred method since it significantly preserves caffeine and chlorogenic acid content while resulting in lower HMF content, and is a method easily implemented at industrial scale. Additionally, the ABTS assay showed no significant difference in antioxidant capacity between oven drying and freeze-drying (*p* > 0.05).

Regarding the antioxidant capacity of melanoidin fractions, CT dried in the oven showed the highest values, followed by freeze-dried CT, sun-dried CT, and CN (*p* < 0.05) in both fractions. Therefore, oven drying represents the best compromise among bioactive compound preservation, food safety, and industrial feasibility. This contrasts with freeze-drying (which is more expensive and has a greater environmental impact) and sun drying (which is more variable and presents hygienic limitations; see Table 6). Oven drying adequately preserves polyphenols and antioxidants, reduces HMF content, and complies with microbiological regulations (Table 6).

Table 4 shows the color measurements for the obtained coffee silverskin. The color is influenced by moisture content. In the case of CT dried by sun dehydration, the process involved a higher temperature (79.1 °C) over a shorter time period, in contrast to oven drying, which involved a longer process at a lower temperature (40 °C). When it comes to CT, changes were observed depending on the drying method: the values of a* and b* increased in the CT (freeze-dried) and CT (oven-dried) samples, while no significant changes were seen in CT (sun-dried). This may be due to the thermal processing effect, moisture loss, and/or the development of Maillard reactions and the formation of colored compounds such as melanoidins. Regarding L*, all values increased after drying; however, CT (sun-dried) was the darkest (lowest L*), which may be attributed to its 5 h treatment inside the sun dryer, reaching up to 79.1 °C. This likely promoted the Maillard reaction, resulting in a higher amount of colored compounds. Among all the dried silverskins, CN was the lightest (highest L*), with higher a* and b* values, indicating a lower degree of Maillard reaction. This could be related to the processing conditions or the absence of a drying step after roasting (see Table 3).

### 3.2. Evaluation of the Extracts for Use as Colorants

**Figure 3:**
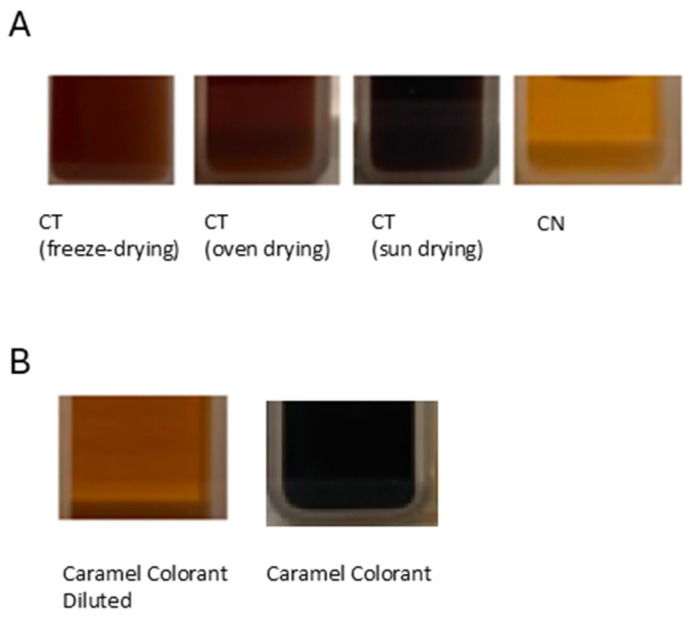
Color comparison of silverskin extract solutions (7 mg/mL) (**A**) and caramel colorant solution (0.7 mg/mL and 7 mg/mL, respectively) (**B**).

Table 5 shows that the torrefacto silverskins have a similar extract yield, while the natural silverskin has a higher yield. As color evaluation suggests (L*, a*, b*, hue, chroma) of the extracts at a concentration of 7 mg/mL (Table 5 and Figure 3), all solutions could serve as potential caramel-like colorants due to the Maillard and caramelization reactions that occur during the roasting process. There are, however, differences among them: the extract from CT (sun) resulted in a darker solution (lower L*) with less color contribution (lower a* and b*), and high absorbance at 420 nm. In particular, the CT (freeze-dried) and CT (oven-dried) appeared very similar in color characteristics (L*, a*, b*, chroma, hue angle). Further, CN produced the lightest solutions (higher L*), but with greater contributions to a*, b*, chromaticity, and hue angle. Regarding coloring capacity, when compared to a caramel colorant, the silverskin with the highest coloring power per gram was the sun-dried silverskin, which could be attributed to oxidation and polymerization of phenolic compounds (which show lower antioxidant activity—see Table 3) due to the higher drying temperature (79.1 °C).

**Figure 4:**
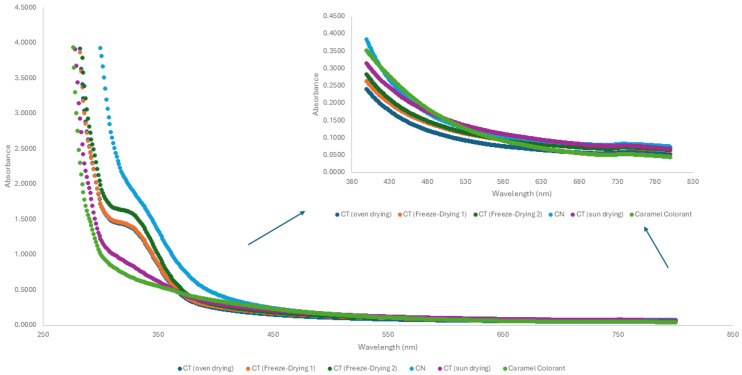
UV-Vis spectra of coffee silverskin extracts and commercial caramel colorant.

Figure 4 shows the UV-visible spectra of the extracts. These exhibit a maximum absorbance near 330 nm, which may be attributed to chlorogenic acid as well as melanoidins [1,35]. In the near-visible UV region and in the visible region, the extracts display similar spectra among themselves and to the caramel colorant.

These color differences can be attributed to the various types of brown compounds, particularly melanoidins, formed during the different processing methods. As shown in Table 3, the sample CT (sun) exhibited the highest content of high molecular weight melanoidins (>10 kDa) (*p* < 0.05), while CT (freeze-dried) and CT (oven-dried) showed statistically similar values (*p* > 0.05). In contrast, CN had the lowest content. These findings align with the color results, where the darkest solution came from CT (sun), likely due to a more advanced Maillard reaction induced by its drying conditions. This also explains the similar coloration observed in CT (oven) and CT (freeze-dried), and the significantly lighter color in CN.

**Figure 5:**
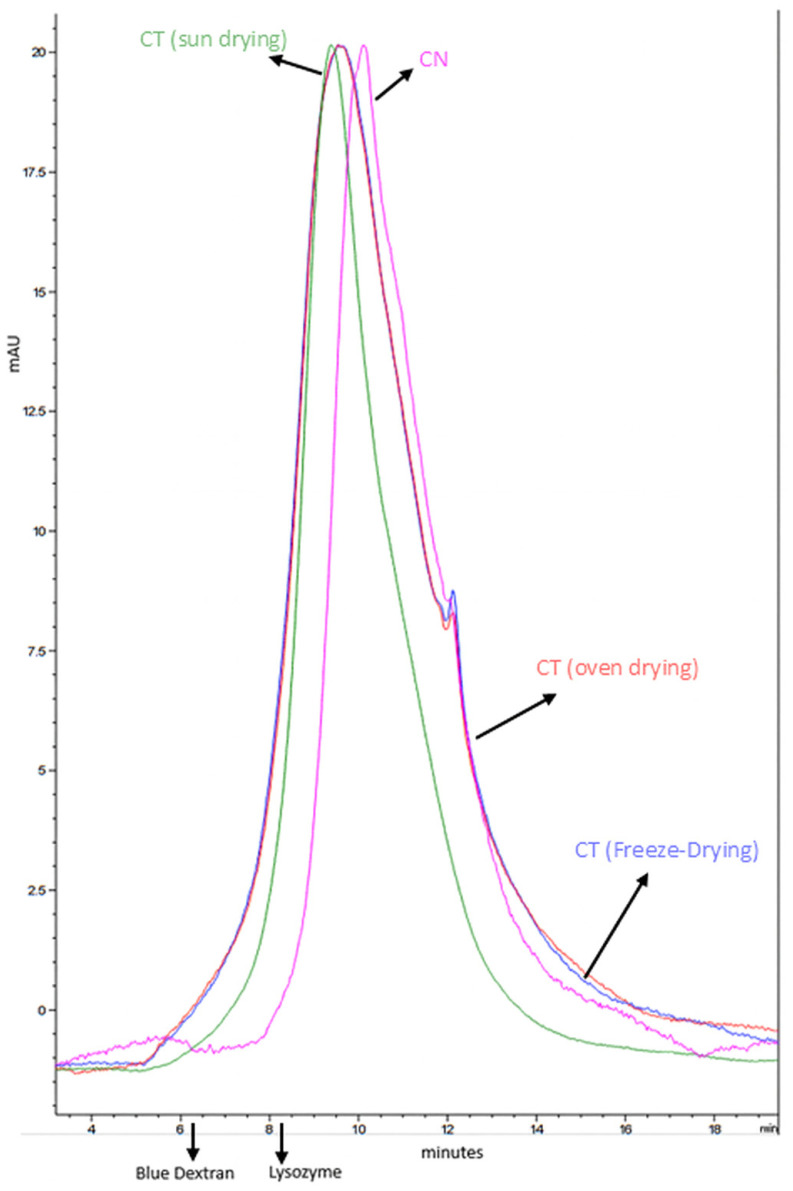
Molecular weight distribution of melanoidins in coffee silverskin at 420 nm.

Further support comes from the molecular weight distribution profiles shown in Figure 5. While all profiles are similar, CT (sun) and CN display narrower peak ranges, which suggests lower molecular diversity. Based on retention times, CT (sun) appears to contain higher molecular weight melanoidins than CN, which is consistent with the ultracentrifugation results and the observed darker color in CT (sun). Similarly, the profiles of CT (freeze-dried) and CT (oven-dried) are closely aligned, which matches the similar intermediate coloration of their solutions between CT (sun) and CN. The presence of a higher content of high-molecular-weight melanoidins is particularly beneficial, not only for enhancing color but also for their potential bioactive properties, as previously discussed.

HMF, a potentially toxic compound, was also evaluated in the extracts. Table 3 shows that all samples had significantly different HMF levels (*p* < 0.05). The highest concentration was found in CN, while CT (sun) had the lowest. Since HMF is an intermediate in the Maillard reaction, its lower presence in CT (sun) may indicate a more complete conversion into melanoidins, which is also supported by its higher color intensity and melanoidin content. However, it is important to note that CT (sun) processing, under the given conditions, also resulted in degradation of free phenolic compounds. Therefore, the resulting color could largely stem from the polymerization of these phenolics, not only from Maillard products.

The Codex Alimentarius (FAO/WHO) Standard for Honey [53] sets a maximum of 40 mg/kg HMF for most honeys and 80 mg/kg for honeys of declared tropical origin. However, Codex does not specify an HMF limit for coffee or coffee by-products, like the CS. Additionally, to the best of our knowledge, no studies have reported acrylamide or HMF levels in coffee silverskin derived from torrefacto roasting. Available data refer only to conventional roasting, with acrylamide values of 152 µg/kg and 161 µg/kg for Arabica and Robusta samples, respectively, and HMF levels below the LOQ [9]. In torrefacto, sugar is added at the final stage of roasting [29,54] favoring the generation of melanoidins and dark color. However, acrylamide formation is known to peak at early roasting stages and may decrease upon prolonged heating [55]. Therefore, the net impact of torrefacto conditions on acrylamide in silverskin cannot be assumed without direct analytical data. On the other hand, the evaluation of HMF in biscuits in biscuits formulated with coffee silverskin showed markedly lower concentrations compared with the sucrose control. While the sucrose biscuits reached 6.51 ± 0.71 mg/kg dry weight, the addition of coffee silverskin reduced HMF levels to a range of 1.65 ± 0.05 to 4.18 ± 0.05 mg/kg dry weight, depending on the amount incorporated. Even at the highest silverskin concentration, HMF values were approximately 36% lower than those in the sucrose biscuits. These levels are also well below the European Food Safety Authority (EFSA) reference range for HMF in biscuits (5–25 mg/kg), confirming the potential of CS as a functional ingredient that contributes to reducing process contaminants during baking [21].

When compared with published data on caramel colorants, which are widely used as food additives, the levels of acrylamide and HMF observed in coffee silverskin are not higher and in many cases considerably lower. Chen et al. [56] reported acrylamide values of ≈20 µg/L in ammonia caramel model systems, while Sung et al. [57] observed acrylamide up to several hundred µg/kg and HMF in the order of hundreds to thousands of mg/kg in non-centrifugal cane sugar with caramel addition. In contrast, acrylamide in our silverskin samples remained well below the EU indicative values and lower than those typically reported for instant coffee (up to 900 µg/kg), and HMF was either absent or below quantification. This comparison indicates that CS, when properly processed, does not pose a higher contaminant burden than caramel colorants already approved and widely used in the food industry.

The absence of published information highlights an important research gap. As a technological strategy to valorize coffee silverskin while minimizing safety risks, melanoidin-rich fractions could be separated using established industrial processes such as aqueous extraction followed by ultrafiltration or chromatographic techniques, which have already been applied to coffee by-products [17,58]. Although such fractionation may increase production costs, it offers a feasible route to obtain a natural colorant or antioxidant ingredient with improved safety and quality assurance.

Based on these findings, CT (sun) appears to be the most promising silverskin for use as a natural colorant, offering a darker color, a higher content of high-molecular-weight melanoidins, and the lowest HMF level. This makes it a strong candidate for use as a food ingredient or coloring agent. From an environmental perspective, sun drying has the lowest overall impact but requires more land use. Optimization through controlled conditions or integration with renewable energy is recommended for industrial applications. Nevertheless, CT (oven-dried) stands out as the most balanced option for functional and technological food uses. It acts both as a source of natural antioxidants and a clean-label caramel-like colorant, offering a dual function not found in traditional caramel color (which serves only as a colorant and preservative). Additionally, it offers an intermediate environmental footprint and a more favorable safety profile.

In this study, the term “clean-label” refers to ingredients that are natural, minimally processed, and free from synthetic additives. CT meets these criteria due to its natural origin, absence of chemical processing, and dual functionality as a colorant and antioxidant source. Its low HMF content further supports its safety profile. While HMF was used as a key indicator, additional parameters such as microbiological safety, melanoidin content, and antioxidant capacity reinforce its suitability for clean-label applications. Regulatory acceptance will require further validation, including consumer perception studies and toxicological assessments.

The results obtained provide key evidence to support the consideration of coffee silverskin as a novel food, facilitating the adoption of coffee by-products in reformulated products rich in fiber and antioxidants.

### 3.3. Microbiological and Toxicological Analysis

For the microbiological characterization of silverskins obtained from the glazing process, only the oven-dried CT was used, as its results were very similar to those of the freeze-dried sample. In contrast, the sun-dried silverskin was excluded due to some outlier results and the high dependency of this drying method on seasonal weather conditions. Additionally, CT (sun) showed the lowest antioxidant capacity, attributed to the degradation of the free phenolic compounds (Table 6).

There is currently no specific regulation for microbiological contaminants in coffee silverskin, so the existing standards for roasted and ground coffee are used as reference. The values obtained fall within local regulatory limits. In Uruguay, the maximum allowed level of ochratoxin A in roasted coffee is 10 µg/kg [59]. As an additional reference, the U.S. Food and Drug Administration (FDA) sets a maximum limit of 20 µg/kg of ochratoxin A in roasted coffee. Differences in OTA content are more plausibly linked to post-roasting handling practices. Torrefacto silverskin is commonly moistened to prevent combustion, which creates a humid environment favorable for fungal development, while natural roast silverskin is usually handled in dry conditions. This suggests that handling practices, rather than botanical origin, are the main factor influencing OTA risk. Regarding mesophilic aerobic bacteria, the Uruguayan National Food Code sets a maximum limit of 10^5^ CFU/g in roasted coffee. The CN (natural silverskin) does not meet this limit, likely due to the absence of a thermal drying treatment, unlike the oven-dried CT. Therefore, a thermal drying step would be necessary to comply with microbiological regulations. According to Regulation (EC) No. 2073/2005 of the European Commission [60], the maximum limit for yeasts and molds in roasted coffee is 10^4^ CFU/g. In Uruguay, Decree No. 266/019 [59] establishes general limits for yeasts and molds in food products, but does not specify a limit for coffee. However, 10^4^ CFU/g is generally accepted. Both the oven-dried CT and CN fall within this parameter.

Thus, the choice of silverskin depends on the intended application. For functional products with antioxidant activity, high bioactive content, and added sensory value (color, flavor), the oven-dried torrefact silverskin (CT-oven) would be the most suitable. It offers the most complete profile for functional food applications at industrial scale, with low cost and scalability. It provides an optimal balance between preservation of functional compounds (thermally stable bioactives such as phenols, caffeine, chlorogenic acid, antioxidants, melanoidins), reduction in undesirable compounds (HMF), and microbiological compliance. It also contains a high concentration of high molecular weight melanoidins and strong coloring power, making it a potential substitute for Class IV caramel coloring. Therefore, CT (oven) could be considered a multifunctional food ingredient for clean-label applications as a natural caramel-like colorant with antioxidant activity, suitable for use in beverages, functional snacks, cereals, baked goods, or dark sauces, offering sustainability, health benefits and food safety. For uses that prioritize food safety without thermal processing and a lighter color, the natural silverskin (CN) may be more appropriate. If environmental impact is the key concern, controlled and optimized sun drying may be explored as a future alternative.

### 3.4. Environmental Impacts of Drying Methods

Selecting an appropriate drying method for coffee silverskin is fundamental not only for preserving its bioactive compounds and ensuring food safety but also for minimizing its environmental footprint. As emphasized by the principles of the circular economy, the valorization of agro-industrial by-products such as CS requires a holistic approach that considers the sustainability of the entire process, including the energy-intensive drying stage. This section presents a comparative Life Cycle Assessment (LCA) of three common drying techniques: oven drying, freeze-drying, and sun drying, with the aim of identifying the most environmentally friendly option. LCA is a widely applied and ISO-standardized methodology [61,62]. It provides quantitative data across multiple environmental impact categories, offering a comprehensive evaluation to identify opportunities for process improvement and sustainability enhancement. The LCA results, based on the ILCD 2011 Midpoint+ calculation method [63], highlight the environmental impacts of the three drying processes used to obtain natural food colorants from CS. The selected functional unit (FU) was 100 g of dried extract.

The LCA results revealed distinct environmental profiles for each drying method. Freeze-drying exhibited the highest environmental impact across several categories, primarily due to its substantial energy consumption. Energy consumption is the main cause associated with the environmental impact of drying process, which is in alignment with other studied drying processes [64,65]. The low temperatures and vacuum conditions necessitate specialized and energy-intensive equipment, leading to a significant carbon footprint. This aligns with general findings in the literature [63], where freeze-drying of food matrices is often associated with high energy demands and greenhouse gas emissions.

In contrast, oven drying of CT demonstrated a considerably lower environmental impact compared to freeze-drying in our assessment. While the specific energy consumption of the oven drying process depends on operational parameters, our findings suggest it is a more energy-efficient alternative for CT. Importantly, as discussed in Section 3.2, the oven-dried CT retained a significant portion of its antioxidant capacity, indicating that the lower energy input did not drastically compromise this key functional property. This balance between energy efficiency and preservation of bioactivity positions oven drying as a potentially sustainable method for CT valorization.

**Figure 6:**
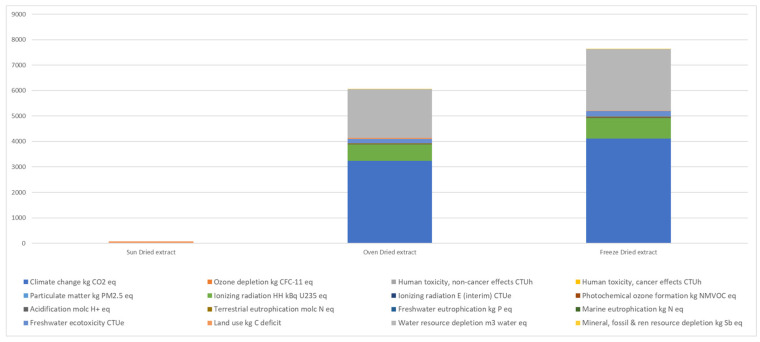
LCA comparison of environmental impact categories for the three drying methods per 100 g of dried extract, assessed using the ILCD 2011 Midpoint+ method.

Sun drying showed no measurable impact across most ILCD 2011 midpoint categories except for land use (Figure 6), which was highest at 78.93 kg C deficit, due to the extended surface area and time required. In contrast, freeze drying exhibited the highest impact values across nearly all categories, with climate change potential reaching 4110 kg CO_2_ eq, water depletion at 2410.75 m^3^, and substantial contributions to toxicity and radiation categories, indicating its heavy energy and resource demands. Oven drying, while less efficient than sun drying in terms of environmental burden, presented a more moderate profile, with climate change impact at 3240 kg CO_2_ eq and lower values across most other indicators compared to freeze drying. Notably, land use impact decreased significantly with increased technological intervention, from sun to oven to freeze drying. However, the overall environmental performance of solar drying is influenced by factors such as the efficiency of the solar drying system, the materials used in its construction, and the potential need for supplementary energy in fluctuating weather conditions. These findings reinforce the trade-off between low-impact but low-control sun drying and high-control but high-impact freeze drying, with oven drying offering a viable middle ground for sustainable industrial application. While our study highlights the potential environmental advantages of sun drying for CS, its practical application may be geographically dependent and will likely require optimization to ensure consistent product quality and drying times.

The environmental assessment identifies sun drying as the most sustainable method, but its limitations in process control and land use underscore the need for further optimization. Oven drying, while associated with a higher environmental footprint, offers a compelling compromise when antioxidant functionality is prioritized. Numerous studies have emphasized the trade-offs between energy intensity and product quality across various drying methods. Our findings are consistent with those of Prosapio et al. [63], who noted that optimizing thermal drying parameters can enhance the retention of bioactive compounds while reducing environmental impact. Similarly, Kumar et al. [66] reported that heat pump drying represents a lower-impact alternative for processing tomato by-products. Furthermore, our previous research suggests that oven drying produces a pigment profile with sensory and structural similarities to caramel, supported by the formation of melanoidins, compounds known for their antioxidant potential [67]. This enrichment aligns with observations by Iriondo DeHond et al. [1], who found that thermal processing enhances antioxidant activity in coffee by-products through the generation of Maillard reaction products. The contribution of melanoidins to both color and bioactivity has been widely discussed and supports their potential application as multifunctional food ingredients.

Our life cycle assessment (LCA) of CT drying methods adds specific, quantitative data to this broader discussion. While freeze-drying offers excellent preservation of certain compounds, its environmental cost may be prohibitive for large-scale implementation, particularly when non-renewable energy sources are used. Sun drying remains the most environmentally benign option in terms of energy consumption but may face challenges in terms of reliability and scalability. Based on our results, conventional oven drying emerges as a promising middle ground: it presents a lower environmental impact than freeze-drying while effectively preserving antioxidant capacity, a key characteristic for its potential application as a functional food ingredient or a source of bioactive compounds. These environmental considerations are essential when evaluating the overall sustainability of CT valorization. The potential of oven-dried CT to serve as a substitute or analog for caramel with added antioxidant properties—as discussed in Section 3.2 and supported by the preservation of melanoidins [17,48]—further reinforces the suitability of this drying method. When both environmental benefits and functional attributes of the final product are taken into account, oven drying presents a particularly promising option.

While this study focused on a gate-to-gate LCA of the drying stage, we acknowledge that a broader cradle-to-grave perspective encompassing the entire coffee processing chain—from cultivation to final product—would provide a more comprehensive understanding of environmental impacts. Future research will aim to integrate upstream agricultural practices, transportation, and downstream food formulation to better assess the full sustainability profile of coffee silverskin valorization.

Future research should explore alternative drying technologies such as sun ovens, flat-plate sun collector dryers, or even traditional ovens powered by fully renewable energy sources, including sun, geothermal, or wind energy. Indeed, these alternative drying methods have been shown to yield higher antioxidant contents than conventional open-sun drying [68]. Additionally, further studies should focus on the detailed characterization of melanoidin fractions, particularly their contribution to antioxidant capacity and color stability. Such analyses would provide stronger evidence to support the positioning of oven-dried coffee silverskin extracts as sustainable caramel analogs with added health benefits.

## 4. Conclusions

This study provides novel insights into the valorization of coffee silverskin (CS) as a sustainable food ingredient, emphasizing the crucial influence of processing method (torrefacto vs. natural) and botanical origin (Coffea arabica vs. Arabica–Robusta blend) on its functional and technological properties.

Torrefacto CS (CT), derived from a blend of Arabica and Robusta beans, exhibited significantly higher concentrations of caffeine, chlorogenic acid, proteins, and melanoidins than natural CS (CN), which originated exclusively from Arabica. These compositional differences position CT as a superior candidate for functional food applications.

Among the drying methods evaluated, oven drying emerged as the most advantageous, offering an optimal balance between preservation of bioactive compounds, microbiological safety, scalability, and moderate environmental impact. While freeze-drying effectively retained bioactivity, its substantial ecological footprint limits industrial viability. Sun drying, although environmentally favorable, presented microbiological challenges and high variability in results.

The oven-dried CT variant demonstrated the greatest potential as a clean-label, bioactive-rich colorant, with melanoidin profiles and coloring power comparable to commercial caramel colorants, alongside recognized health-promoting properties. Its functional benefits, combined with safe compositional and microbial parameters, support its application in food reformulation strategies aligned with clean-label and circular economy principles.

Considering the environmental impact of thermal processes, further research should focus on integrating renewable energy sources, such as sun thermal or wind power, to operate industrial ovens, thereby reducing the carbon footprint of CS processing and enhancing overall sustainability.

Overall, while heat-induced contaminants in conventional silverskin appear within acceptable limits, the main bottleneck for valorization remains microbiological safety, since husks are often moistened during storage to prevent combustion. Establishing standardized drying and hygienic management protocols is essential to ensure its suitability as a food-grade ingredient. In addition, the use of fractionation strategies to recover melanoidin-rich fractions offers a feasible albeit cost-increasing route for producing natural colorants or antioxidant additives.

These findings contribute to the scientific foundation for recognizing coffee silverskin, particularly torrefacto CS (CT), as a novel food ingredient. Furthermore, they support industry-wide transitions toward zero-waste practices, functional innovation, and climate-resilient food systems, thereby advancing the achievement of Sustainable Development Goals (SDGs). The contribution of this study to the SDGs can be supported by quantitative indicators. For SDG 9 (Industry, Innovation, and Infrastructure), the valorization of coffee silverskin through scalable oven drying represents a sustainable industrial innovation. For SDG 12 (Responsible Consumption and Production), the use of a food-grade by-product aligns with circular economy principles and waste reduction. For SDG 13 (Climate Action), our LCA results show a 19% reduction in greenhouse gas emissions when using oven drying compared to freeze-drying, alongside lower water depletion and toxicity impacts. These metrics provide measurable evidence of progress toward sustainability targets.

## Figures and Tables

**Table 1:** Composition of different coffee types: Arabica and Robusta (adapted from Illy & Viano, 2005 [10]).

	Content (% Dry Basis)	
Component	Arábica	Robusta
Soluble carbohydrates	9–12.5	6–11.5
Insoluble carbohydrates	46–53	34–44
Lipids	15–18	12–18
Protein	8.5–12	8.5–12
Chlorogenic acid	6.7–9.2	7.1–12.1
Caffeine	0.8–1.4	1.7–4.0

**Table 2:** Caffeine and chlorogenic acid contents obtained by different extraction methods performed on lyophilized CT.

Extraction Method	Chlorogenic Acid (ppm)	Caffeine (ppm)
Water at 100 °C 10 min, (500 mg, 10 mL)	2962 ± 324 ^b^	5877 ± 417 ^b^
1% Aqueous formic acid at 70 °C 1 h, (5 mL, 200 mg)	1780 ± 27 ^c^	3895 ± 46 ^c^
**EtOH:Water 1:1 at 40 °C for 60 min (25 mL, 500 mg)**	**3327 ± 183 ^a^**	**7198 ± 395 ^a^**
MeOH:Agua 70:30, (1 g, 10 mL)	2381 ± 66 ^b^	5240 ± 77 ^b^

Different superscript letters in a column indicate significant differences (*p* < 0.05).

**Table 3:** Characterization of coffee silverskin samples from different origins and drying methods (dry basis).

	CT (Freeze-Dried)	CT (Oven-Dried)	CT (Sun-Dried)	CN
Fiber (%)	66.22 ± 0.28 ^a.b^	67.25 ± 0.61 ^a^	63.79 ± 1.01 ^b^	66.50 ± 0.89 ^a.b^
Protein (%)	15.68 ± 0.68 ^a^	15.69 ± 0.22 ^a^	15.48 ± 0.42 ^a.b^	14.23 ± 0.28 ^b^
Lipids (%)	3.32 ± 0.29 ^a.b^	2.72 ± 0.14 ^b^	3.04 ± 0.11 ^a.b^	4.81 ± 0.51 ^a^
Ashes (%)	6.63 ± 0.56 ^a^	6.93 ± 0.46 ^a^	6.93 ± 0.68 ^a^	6.43 ± 0.61 ^a^
Carbohydrates (by difference) (%)	8.16	7.41	10.76	8.03
Total Phenolics (mg GAE/g silverskin)	25.14 ± 0.92 ^a^	27.02 ± 0.51 ^a^	26.63 ± 1.06 ^a^	26.63 ± 0.79 ^a^
TEAC (µmol TE/g silverskin)	105.98 ± 0.47 ^a^	95.53 ± 0.39 ^a^	16.72 ± 0.85 ^c^	52.10 ± 0.62 ^b^
Caffeine (ppm)	7198 ± 395 ^a^	8097 ± 293 ^a^	1419 ± 14 ^c^	5657 ± 234 ^b^
Chlorogenic Acid (ppm)	3327 ± 183 ^a^	3016 ± 85 ^a^	60 ± 4 ^c^	616 ± 95 ^b^
Melanoidins (% PM > 10 kDa/total)	33.80 ± 1.02 ^b^	30.49 ± 1.15 ^b^	38.53 ± 0.72 ^a^	19.66 ± 0.54 ^c^
TEAC (µmol TE/g melanoidins PM > 10 kDa)	592.71 ± 10.12 ^b^	627.76 ± 11.56 ^a^	445.09 ± 7.75 ^c^	178.87 ± 9.22 ^d^
TEAC (µmol/g melanoidins PM < 10 kDa/total)	770.59 ± 68.19 ^b^	1028.63 ± 51.11 ^a^	538.47 ± 32.19 ^c^	419.42 ± 27.81 ^d^
HMF (ppm)	14 ± 0.7 ^b^	4 ± 0.07 ^c^	1 ± 0.04 ^d^	35 ± 1 ^a^

Different letters in a row indicate significant difference (*p* < 0.05). TE: Trolox equivalent; GAE: Gallic Acid Equivalent; TEAC: Trolox Equivalent Antioxidant Capacity.

**Table 4:** Values of L*, a*, and b* values for solid samples. The dE*ab was calculated for the CT samples in relation to the wet silverskin (prior to drying).

Sample	L*	a*	b*	dE*ab	Digital Color Image
CT (wet)	38.30 ± 2.29 ^c^	1.20 ± 1.14 ^d^	2.08 ± 0.25 ^d^	-	
CT (oven-dried)	43.81 ± 1.91 ^b^	3.05 ± 0.22 ^c^	6.32 ± 0.59 ^c^	7.34 ^b^	
CT (sun-dried)	42.79 ± 0.26 ^b^	1.36 ± 0.11 ^d^	2.49 ± 0.23 ^d^	4.52 ^c^	
CT (freeze-dried)	47.84 ± 1.58 ^a^	3.71 ± 0.39 ^b^	8.92 ± 0.57 ^b^	12.03 ^a^	
CN	46.82 ± 0.85 ^a^	7.52 ± 0.23 ^a^	23.13 ± 0.53 ^a^	-	

CT wet is raw CT prior to drying. Different letters within the same column indicate significant differences (*p* < 0.05).

**Table 5:** Yield of extraction of colored compounds, coloring power relative to a caramel colorant for the different silverskins, and color values (L*, a*, b*, h*ab, C*ab) and absorbance at 420 nm.

			Extracts (7 mg/mL)					
Extract	Yield (% mg Extract/mg Silverskin)	mg eq Colorant/g Silverskin	L*	a*	b*	h*ab	C*ab	Absorbance at 420 nm
CT (oven-dried)	5.86	300.86 ^c^	12.51	16.88	15.76	43.03	23.09	1.2153
CT (sun-dried)	6.05	455.27 ^a^	6.79	6.35	4.13	33.04	7.57	1.6264
CT (freeze-dried)	6.18	374.33 ^b^	13.74	13.91	15.44	47.97	20.78	1.2646
CN	8.52	123.75 ^d^	47.39	7.24	42.66	80.37	43.27	1.7711
Class IV Caramel Colorant (0.7 mg/mL)			19.51	20.42	27.03	52.93	33.88	1.6951

Different letters within the same column indicate significant differences (*p* < 0.05).

**Table 6:** Microbiological and toxicological analysis of CT (oven-dried) and CN silverskin samples.

Test/Assay	CN	CT (Oven)
Ochratoxin A	6.2 µg/kg	3.9 µg/kg
Aerobic Mesophilic Count at 30 °C	2.9 × 10^6^ ufc/g	8.3 × 10^3^ ufc/g
Mold Count	<10 ufc/g	7.7 × 10^2^ ufc/g
Yeast Count	10 ufc/g	3.9 × 10^3^ ufc/g

Only the most representative treatments are included; see text for rationale.

## Data Availability

The raw data supporting the conclusions of this article will be made available by the authors on request.

## Abbreviations

The following abbreviations are used in this manuscript:

CS	Coffee silverskin
CT	Torrefacto
CN	Natural
HMF	5-hydroxymethylfurfural
SDG	Sustainable Development Goals
LCA	Life Cycle Assessment
